# Association of the systemic immune-inflammation index (SII) and clinical outcomes in patients with stroke: A systematic review and meta-analysis

**DOI:** 10.3389/fimmu.2022.1090305

**Published:** 2022-12-15

**Authors:** Yong-Wei Huang, Xiao-Shuang Yin, Zong-Ping Li

**Affiliations:** ^1^ Department of Neurosurgery, Mianyang Central Hospital, School of Medicine, University of Electronic Science and Technology of China, Mianyang, Sichuan, China; ^2^ Department of Immunology, Mianyang Central Hospital, School of Medicine, University of Electronic Science and Technology of China, Mianyang, Sichuan, China

**Keywords:** systemic immune-inflammation index, stroke, SII, clinical outcome, meta-analysis

## Abstract

**Introduction:**

A novel systemic immune-inflammation index (SII) has been proven to be associated with outcomes in patients with cancer. Although some studies have shown that the SII is a potential and valuable tool to diagnose and predict the advise outcomes in stroke patients. Nevertheless, the findings are controversial, and their association with clinical outcomes is unclear. Consequently, we conducted a comprehensive review and meta-analysis to explore the relationship between SII and clinical outcomes in stroke patients.

**Methods:**

A search of five English databases (PubMed, Embase, Cochrane Library, Scopus, and Web of Science) and four Chinese databases (CNKI, VIP, WanFang, and CBM) was conducted. Our study strictly complied with the PRISMA (the Preferred Reporting Items for Systematic Reviews and Meta-Analyses). We used the NOS (Newcastle-Ottawa Scale) tool to assess the possible bias of included studies. The endpoints included poor outcome (the modified Rankin Scale [mRS] ≥ 3 points or > 3 points), mortality, the severity of stroke (according to assessment by the National Institute of Health stroke scale [NIHSS] ≥ 5 points), hemorrhagic transformation (HT) were statistically analyzed.

**Results:**

Nineteen retrospective studies met the eligibility criteria, and a total of 18609 stroke patients were included. Our study showed that high SII is significantly associated with poor outcomes (odds ratio [OR] 1.06, 95% confidence interval [CI] 1.02-1.09, P = 0.001, I^2^ = 93%), high mortality (OR 2.16, 95% CI 1.75-2.67, P < 0.00001, I^2^ = 49%), and the incidence of HT (OR 2.09, 95% CI 1.61-2.71, P < 0.00001, I^2^ = 42%). We also investigated the difference in SII levels in poor/good outcomes, death/survival, and minor/moderate-severe stroke groups. Our analysis demonstrated that the SII level of the poor outcome, death, and moderate-severe stroke group was much higher than that of the good outcome, survival, and minor stroke group, respectively (standard mean difference [SMD] 1.11, 95% CI 0.61-1.61, P < 0.00001 [poor/good outcome]; MD 498.22, 95% CI 333.18-663.25, P < 0.00001 [death/survival]; SMD 1.35, 95% CI 0.48-2.23, P = 0.002 [severity of stroke]). SII, on the other hand, had no significant impact on recanalization (OR 1.50, 95% CI 0.86-2.62, P = 0.16).

**Discussion:**

To the best of our knowledge, this may be the first meta-analysis to look at the link between SII and clinical outcomes in stroke patients. The inflammatory response after a stroke is useful for immunoregulatory treatment. Stroke patients with high SII should be closely monitored, since this might be a viable treatment strategy for limiting brain damage after a stroke. As a result, research into SII and the clinical outcomes of stroke patients is crucial. Our preliminary findings may represent the clinical condition and aid clinical decision-makers. Nonetheless, further research is needed to better understand the utility of SII through dynamic monitoring. To generate more robust results, large-sample and multi-center research are required.

**Systematic review registration:**

https://www.crd.york.ac.uk/prospero/, identifier CRD42022371996.

## Introduction

Cerebrovascular disease is the second majority cause of death and disability worldwide. Stroke, including ischemic and hemorrhagic, is the leading component of it. More than 2.4 million newly diagnosed strokes occur in China yearly, and the mortality rate has risen to 22.3% (1, 2). Of these patients, 87% are ischemic stroke (3). Therefore, assessing stroke patients’ risk and severity early and identifying risk factors that can be addressed through intervention can improve the dismal outcomes for stroke patients (4).

In recent two years, Scholars progressively recognize the secondary injury of the brain’s inflammatory response after stroke. A study by Kim et al. (5) has shown that inhibiting inflammatory cells could alleviate brain injury. Therefore, inflammatory factor-related immunotherapy may become a potential treatment to improve the outcomes of stroke patients (6).

Systemic immune inflammatory index (SII) has been used as a prognostic marker for some diseases. A meta-analysis by Zhang et al. has shown that elevated pretreatment SII was significantly associated with worse overall survival and recurrence-free survival/progression-free survival in with biliary tract cancers (7). SII based on platelets×neutrophils/lymphocytes (P × N/L) was reported to accurately predict outcomes in patients with venous sinus thrombosis (8). Two studies have shown that SII is related to the severity of stroke at admission (9, 10), but this index has no wide application to predict functional outcomes in stroke patients. Although some studies have shown that the SII is a potential and valuable tool to diagnose and predict the advise outcomes in stroke patients. Nevertheless, the findings are controversial, and their association with clinical outcomes is unclear. Consequently, we conducted a comprehensive review and meta-analysis to explore the relationship between SII and clinical outcomes in stroke patients.

## Methods

### Aims and PICO statement

Our study strictly complied with the PRISMA (the Preferred Reporting Items for Systematic Reviews and Meta-Analyses) statement. We registered our study at PROSPERO with the identifier CRD42022371996 (https://www.crd.york.ac.uk/PROSPERO/) (11). The PRISMA checklist is presented in **Supplemental Table 1**. These were the PICO statements: 1) Population: patients who have had an ischemic or hemorrhagic stroke (also known as an ICH). 2) Intervention: mechanical thrombectomy, intravenous thrombolysis, or none of the above. 3) Comparisons: relative low SII *vs.* relative high SII, and the grouping definition is the same as our previous study (12). 4) Outcomes: the mRS ≥ 3 points or > 3 points at follow-up was defined as poor outcome, and we categorized stroke severity as NIHSS≥ 5 points. In AIS patients, symptomatic intracerebral hemorrhage was considered hemorrhagic transformation (HT). Besides, mortality and the SII level of the poor/good outcome and death/survival groups were also extracted.

### Literature search strategy

In order to decrease the selectivity bias, a search of five English databases (PubMed, Embase, Cochrane Library, Scopus, and Web of Science) and four Chinese databases (CNKI, VIP, WanFang, and CBM) was conducted. Two reviewers (Huang YW and Yin XS) systematically screened the databases for the relevant studies published from databases inception to the end of November 2022. The following search strategy was applied: (“systemic immune-inflammation index” OR “SII”) AND (“stroke”) for English databases, and “(主题=全身炎症免疫指数) AND (主题=卒中)” for Chinese databases. The detailed search strategy is presented in **Supplemental Table 2**. We also comprehensively searched the main clinical registry centers such as ClinicalTrials.gov, WHO-ICTRP, and ChiCTR for unpublished works and gray literature in Greynet, OpenSIGLE, and HMIC databases. The purpose is to decrease the publication bias as far as possible.

### Inclusion and exclusion criteria

After the inclusion and exclusion criteria, all potential studies were appraised independently by two reviewers (Huang YW and Yin XS). The reviewers assessed studies that met all the following criteria: 1) types of publication: articles published publicly without language restriction. 2) types of participants: stroke patients with complete data. 3) types of comparison: relative low SII *vs.* relative high SII. 4) types of outcome measure: poor outcome, the severity of the stroke, mortality, HT, and the SII level of poor/good outcome group, death/survival group. Case reports, reviews, notes, meta-analyses, editorials, letters to the editor, commentaries, and conference abstracts were excluded.

### Data extraction

Two reviewers extracted data independently and used the same tables of data extraction. The extracted data were as follows: 1) essential characteristics: first author name, publication year, nation, study design, and participant count (n); 2) participant characteristics: age(y) (Mean ± SD), male (%), type of stroke, medical history, medication history, time of blood sample, laboratory test method, type of intervention, cutoff of SII, primary endpoints, and clinical follow-up (d); 3) information on interesting results, etc.

### Risk of bias assessment

To evaluate the possible bias of the included research, we used the NOS (Newcastle-Ottawa Scale) method (13). The three aspects of the method based on NOS were described in **Supplemental Table 3** and **Table 1**, together with the specifics and outcomes for each. Studies were scored on a scale of one to nine, with over six scores being regarded to be of excellent quality. The evaluation was carried out separately by three reviewers (Huang YW, Yin XS, and Li ZP). Any disagreement was settled, if necessary, in a group investigation discussion.

**Table 1:** The baseline characteristics of included studies.

Author	Year	Nation	Study Design	Participants(n)	Age (y)(mean ± SD)	Male(%)	Type ofStroke	MedicalHistory	MedicationHistory	Time ofBlood Sample	Laboratory TestMethod	Type ofIntervention	Cutoffof SII	Primary Endpoints	Follow-up (d)	NOS
Chu et al. (14)	2020	Taiwan, China	Retrospective Single-center	415	70.7 ± 13.5	61.69	AIS	①②③ ⑤⑥⑦⑧	–	On arrival in the emergency room	Full blood	–	651.00	PO	–	9
Trifan et al. (15)	2020	America	Retrospective Single-center	239	58.1 ± 3.2	56.90	ICH	①② ⑪⑯⑰	Aspirin Clopidogrel Anti-hypertensive Statins	On arrival in the hospital	–	–	730.00	PO	discharge	8
Hou et al. (9)	2021	China	Retrospective Single-center	362	67.8 ± 12.2	67.40	AIS	①②④	–	The following day (6:00 am), after admission	Full blood	–	–	Stroke severity	–	9
Li et al. (16)	2021	China	Retrospective Single-center	291	57.0 ± 14.0	66.67	ICH	–	–	During hospitalization	–	–	1700.00	PO	90 d	7
Weng et al. (17)	2021	China	Retrospective Single-center	216	67.8 ± 3.5	62.96	AIS	①②③ ④⑤⑨	–	The first 24 h	–	IVT	545.14	PO	90 d	8
Yang et al. (18)	2021	China	Retrospective Single-center	310	65.0 ± 11.4	72.58	AIS	①②⑤⑫	Anti-platelet Anti-coagulation	Next morning (5:00 am) after admission	–	–	653.65	HT	–	9
Yi et al. (19)	2021	Korea	Retrospective Single-center	440	70.0 ± 12.9	58.41	AIS	①②④ ⑤⑨⑫	–	On admission	Peripheral venous blood	MT	853.00	PO	90 d	8
Acar et al. (20)	2022	Turkey	Retrospective Single-center	123	66.5 ± 12.0	52.85	AIS	①②④ ⑤⑨⑫	Anti-platelet Anti-coagulation	At admission to emergency room	Venous blood	EVENT	1690.00	PO Cerebral reperfusion	90 d	8
Adiguzel et al. (21)	2022	Turkey	Retrospective Single-center	205	71.0 ± 15.0	41.46	AIS	①② ④⑱⑲	–	The first 14 days	–	–	–	Mortality PO Pneumonia	90 d	7
Chen et al. (22)	2022	Taiwan, China	Retrospective Single-center	3402	71.9 ± 2.8	57.58	AIS	①②④⑤ ⑥⑦⑧⑫	–	Within 24 h of admission	–	IVT or EVT	2120.00 (IHIS) 1051.00 (OHIS)	Mortality PO	discharge	7
Hsu et al. (23)	2022	Taiwan, China	Retrospective Single-center	374	65.4 ± 17.8	64.44	ICH	①②⑤⑥	–	On arrival at the emergency room	Full blood	–	–	PO Mortality	discharge	8
Huang et al. (10)	2022	China	Retrospective Single-center	234	68.9 ± 3.7	50.43	AIS	①②④	Anti-platelet Anti-coagulation	Within 24 h of admission	Venous blood	–	1008.00	Stroke severity	discharge	9
Ji et al. (24)	2022	China	Retrospective Single-center	675	67.1 ± 11.4	59.56	AIS	①②④	–	Within the first 24h after admission	–	EVENT	2140.00	PO	90 d	9
Wang et al. (25)	2022	China	Retrospective Multi-center	9107	61.9 ± 11.1	69.65	AIS	①②④ ⑨⑩⑯	–	Within 24 h after admission	venous blood	–	–	Mortality PO Recurrent stroke	90 d 1 y	7
Wu et al. (26)	2022	China	Retrospective Multi-center	1181	69.1 ± 15.6	50.80	AIS	①②③ ④⑧⑪⑳	Warfarin NOAH Anti-platelet	The first test after entering the ICU	–	–	–	Mortality	30 d 90 d	7
Yang et al. (27)	2022	China	Retrospective Single-center	379	70.8 ± 3.5	52.51	AIS	①②③④	Statin Anti-thrombotic	On admission	–	IVT & EVT	–	HT	–	8
Zhou et al. (28)	2022	China	Retrospective Single-center	208	63.3 ± 11.3	68.75	AIS	①②④⑮	–	Within 24 h	Routine blood	–	802.8	Stroke severity PO	90 d	9
Zhu et al. (29)	2022	China	Retrospective Single-center	182	39.4 ± 6.8	83.52	AIS	①②	–	Within 24 h of admission	Peripheral venous blood	–	781.40	Stroke severity PO	90 d	9
Liu et al. (30)	2022	China	Retrospective Single-center	266	65.0 ± 10.9	60.15	AIS	①②④	–	Within 24 h of admission	Venous blood	–	728.03 (HT) 449.76 (PO)	HT PO	90 d	9

AIS, acute ischemic stroke; IS, ischemic stroke; ICH, intracerebral hemorrhage; CKD, chronic kidney disease; AF, atrial fibrillation; CAD, coronary artery disease; CHD, coronary heart disease; MI, myocardial infarct; CHF, congestive heart failure; PVD, peripheral vascular disease; CPD, chronic pulmonary disease; UTI, urinary tract infection; EVT, endovascular treatment; IVT, intravenous thrombolysis;

MT, mechanical thrombectomy; IHIS, in-hospital ischemic stroke; NOAC, new oral anticoagulants.OHIS, out-of-hospital ischemic stroke; NOS, Newcastle-Ottawa Scale; HT, hemorrhagic transformation.

PO, poor outcome

①Hypertension; ②Diabetes; ③Hyperlipidemia; ④AF; ⑤Prior stroke; ⑥Heart disease; ⑦Uremia; ⑧Cancer; ⑨CAD; ⑩Hypercholesterolemia; ⑪CKD; ⑫Dyslipidemia; ⑬Uremia; ⑭CAD; ⑮CHD; ⑯IS; ⑰ ICH; ⑱Pneumonia; ⑲UTI; ⑳MI, CHF, PVD, Dementia, CPD.

### Statistical analysis

For dichotomous variables, odds ratios (ORs) and their corresponding 95% confidence interval (CIs) were calculated. Mean difference (MD) and their corresponding 95% CIs were calculated for continuous variables. If the values of some continuous variable varied greatly, we utilized standard mean difference (SMD) to perform the meta-analysis. Besides, we extracted the ORs adjusted by confounding factors and their corresponding 95% CIs in some studies. The confounding factors of each study were provided in **Supplemental Table 4**. We estimated the mean and standard deviation (SD) by the sample size, median, and interquartile range. The optional estimating methods were from Luo et al. (31) and Wan et al. (32). The website is https://www.math.hkbu.edu.hk/~tongt/papers/median2mean.html. To account for clinical heterogeneity, we conducted meta-analyses and subgroup analyses using the random-effects or fixed-effects model (33). The Cochrane Q test was used to evaluate the heterogeneity (P < 0.1 or I2 > 50% was significant heterogeneity) (34). P < 0.05 was statistically significant. Specific data of the high SII and low SII groups were extracted from the studies based on our grouping definition. The publication bias was assessed by funnel plot. We performed the statistical analyses by Review Manager software (version 5.3.3; https://training.cochrane.org/online-learning/core-softwarecochrane-reviews/revman).

## Results

**Figure 1:**
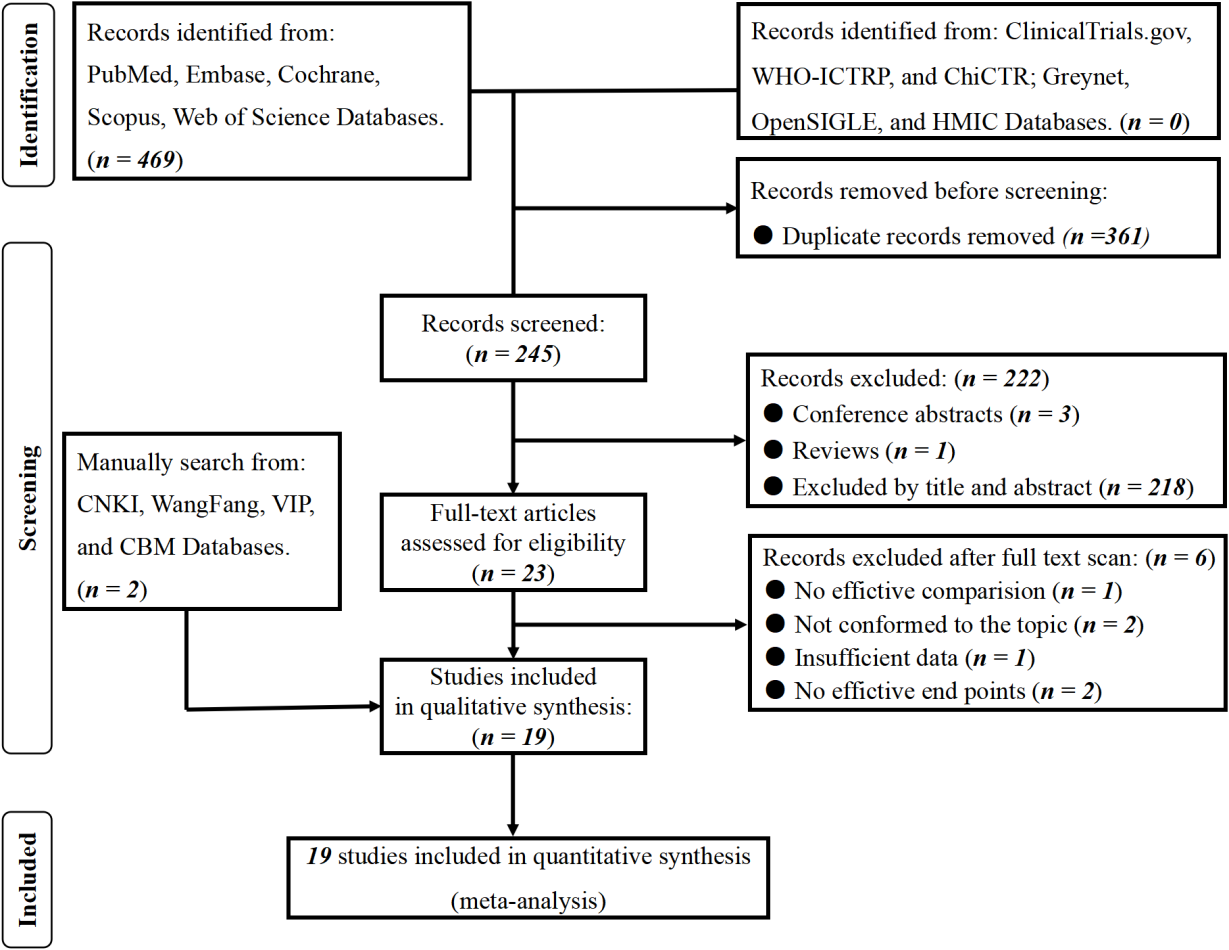
PRISMA flowchart of included studies.

The primary search in English databases yielded 469 records. Two hundred twenty-four duplicates were excluded, and 245 remained. Two hundred twenty-two records were further excluded after title/abstract and publication type screening. Then, 23 potentially eligible articles were retained for full-text assessment, and six were excluded for insufficient data, theme, endpoints, and groupings. Besides, we manually searched the Chinese databases, and two articles met the included criteria. Finally, 19 studies (9, 10, 14–30) met the eligibility criteria, and a number of 18609 stroke patients were involved. All the studies were retrospective. The literature search process is shown in **Figure 1**, and the systematic summary is summarized in **Table 1**.

### Meta-Analysis and subgroup analysis of different outcomes

**Figure 2:**
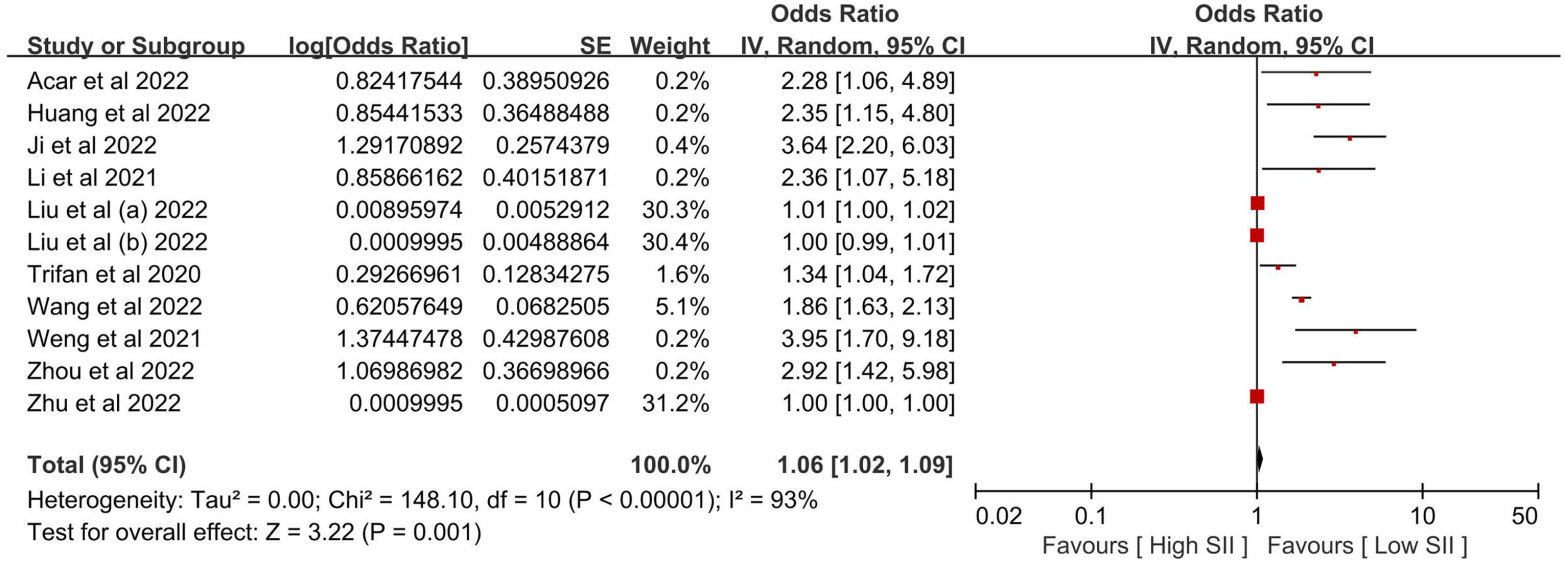
The poor outcome between the high SII and low SII groups. Liu et al. (a) and Liu et al. (b) were from the same study, Liu et al. (c) presented minor stroke patients, and Liu et al. (b) presented moderate-to-severe stroke patients. We regarded the two groupings as independent studies when performing a meta-analysis of poor outcomes.

**Figure 3:**
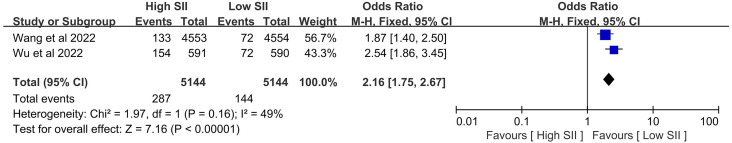
The mortality between the high SII and low SII groups.

**Figure 4:**
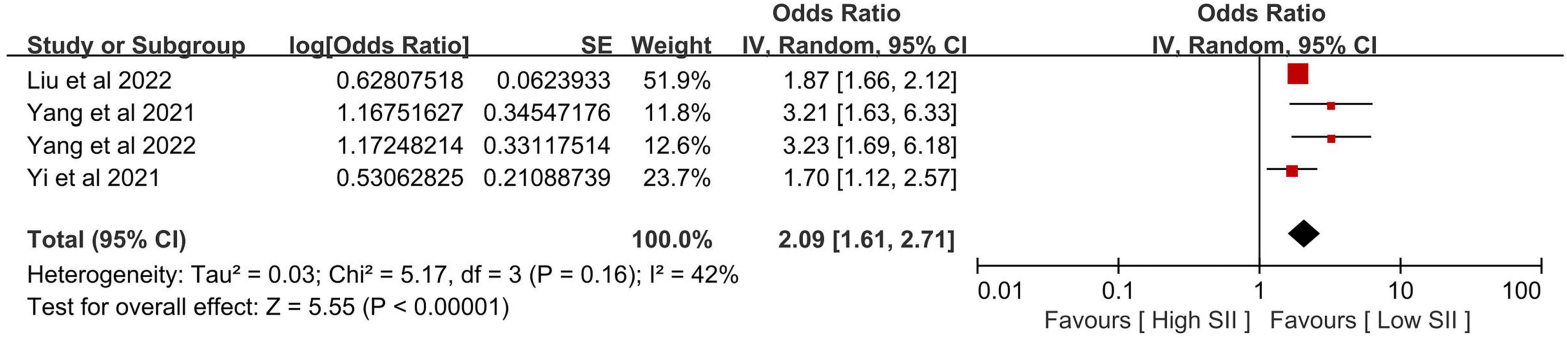
The HT between the high SII and low SII groups.

**Figure 5:**
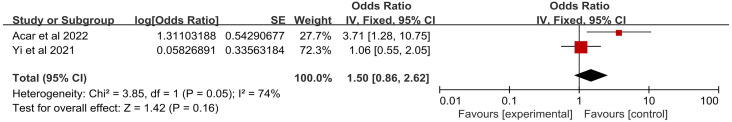
The recanalization between high SII and low SII groups.

**Table 2** provides a summary of the findings. When comparing the poor outcome between the low SII group and the high SII group, our analysis showed that high SII was significantly associated with poor outcome (OR 1.06, 95% CI 1.02-1.09, P = 0.001, I^2^ = 93%; **Figure 2**). When comparing the mortality between the low SII group and the high SII group, our analysis showed that high SII was significantly associated with high mortality (OR 2.16, 95% CI 1.75-2.67, P < 0.00001, I^2^ = 49%; **Figure 3**). When comparing the HT between the low SII group and the high SII group, our analysis showed that high SII was significantly associated with HT (OR 2.09, 95% CI 1.61-2.71, P < 0.00001, I^2^ = 42%; **Figure 4**). SII, on the other hand, had no significant impact on recanalization (OR 1.50, 95% CI 0.86-2.62, P = 0.16; **Figure 5**).

**Table 2:** Meta-Analysis and subgroup analysis of Different Outcomes.

			Results	
Items	Studies, n	OR (95% CI)	*P* Value	Heterogeneity (I2, *P* for Cochran Q)
**Poor Outcome**				
Pooled	11	1.06 (1.02, 1.09)	*P = 0.001*	I2 = 93%, *P < 0.00001*
				
China	9	1.05 (1.01-1.08)	*P = 0.005*	I2 = 94%, *P < 0.00001*
				
Non-China	2	1.54 (0.97-2.43)	*P = 0.06*	I2 = 40%, *P =0.19*
				
AIS	9	1.05 (1.01-1.08)	*P = 0.005*	I2 = 94%, *P < 0.00001*
				
ICH	2	1.56 (0.95-2.57)	*P = 0.08*	I2 = 45%, *P =0.18*
				
NNo Surgery Intervention	8	1.04 (1.01-1.07)	*P = 0.01*	I2 = 94%, *P < 0.00001*
				
IVT, EVT, or MT	3	3.30 (2.27-4.81)	*P < 0.00001*	I2 = 0%, *P =0.54*
				
**Mortality**	2	2.16 (1.75-2.67)	*P < 0.00001*	I2 = 49%, *P* = 0.16
				
**HT**	4	2.09 (1.61-2.71)	*P < 0.00001*	I2 = 42%, *P* = 0.16
				
**Recanalization**	2	1.50 (0.86-2.62)	*P = 0.16*	I2 = 74%, *P* = 0.05
			**Results**	
**Items**	**Studies, n**	**SMD or MD (95% CI)**	***P* Value**	**Heterogeneity (I2, *P* for Cochran Q)**
				
**PPoor outcome / Good outcome**	9	1.11 (0.61-1.61)	*P* < 0.00001	I2 = 98%, *P* < 0.00001
				
**Death / Survival**	3	498.22 (333.18-663.25)*	*P* < 0.00001	I2 = 0%, *P* = 0.68
				
**Severity of Stroke**	3	1.35 (0.48-2.23)	*P* = 0.002	I2 = 96%, *P* < 0.00001

* The SII level did not vary greatly, so we utilized mean difference (MD) to perform the meta-analysis.

**Figure 6:**
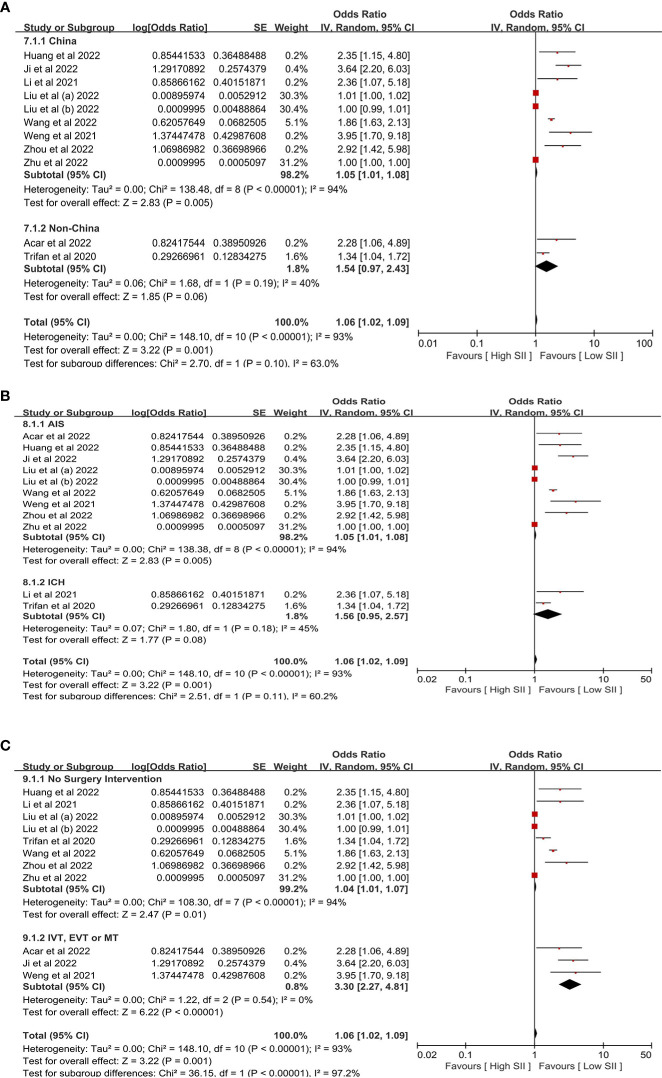
The subgroup analysis of poor outcomes is based on **(A)** different countries, **(B)** different types of stroke, and **(C)** surgery intervention.

Because the other endpoints did not have enough included studies to perform subgroup analysis, we only performed the subgroup analysis of clinical heterogeneity of poor outcomes. In the Subgroup analysis, we identified that the different countries, types of stroke, and surgery intervention (IVT, EVT, or MT) were associated with clinical heterogeneity of poor outcomes (**Figures 6A–C**).

**Figure 7:**
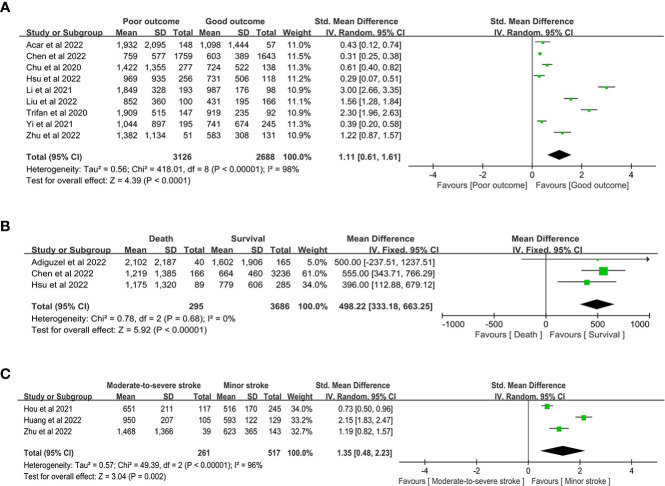
the SII level between the **(A)** poor outcome and good outcome group, **(B)** death and survival group, and **(C)** moderate-to-severe and minor stroke group.

When comparing the SII level between the poor outcome and good outcome groups, our study showed that poor outcomes had higher SII (SMD 1.11, 95% CI 0.61-1.61, *P* < 0.00001; **Figure 7A**). When comparing the SII between the death and survival groups, our study showed that the death group had higher SII (MD 498.22, 95% CI 333.18-663.25, P < 0.00001, I^2^ = 0%; **Figure 7B**). Our study compared the SII between the moderate-to-severe and minor groups and showed that the moderate-to-severe group had higher SII (SMD 1.35, 95% CI 0.48-2.23, P = 0.002; **Figure 7C**).

Some outcomes had substantial heterogeneity, and we could not decrease it by removing studies one by one. Therefore, the source of heterogeneity may be the studies themselves, the study design, the parameter measurement tool, the highly variable duration of follow-up, and so on. Although high heterogeneity influenced the robustness of our results, the preliminary findings were still worth paying attention to.

### Risk of bias assessment and publication bias assessment

The NOS evaluated and assigned a median of 8 stars to all research, with an interquartile of (7-9) stars. The methodological quality of the included studies is displayed in **Supplemental Table 3**. The funnel plot results, which evaluated the probability of publication bias, are shown in **Supplemental Figure 1**.

## Discussion

Exploring the inflammatory response mechanism after stroke is beneficial for immunoregulatory therapy applications (10). The SII, calculated from neutrophils, lymphocytes, and platelets, is more reliable and representative than other leukocyte-based indicators of inflammation, including platelet-to-lymphocyte ratio (PLR) and lymphocyte-to-monocyte ratio (LMR). In addition, SII has the advantage of being easily accessible and rapid, as routine blood analysis is essential for patients admitted to the hospital at no additional cost to the patient, thus improving compliance.

The relationship between high SII levels and the clinical outcome of stroke patients remains unclear. Possible mechanisms are as follows: first, in the leukocyte family of the peripheral circulation, neutrophils first infiltrate the lesion within hours after stroke, further leading to the release of inflammatory mediators that directly cause necrosis and apoptosis of cells in the ischemic zone (35, 36). Neutrophils are an essential source of cytokines, free radicals, and matrix metalloproteinase-9, which induce apoptosis of neuronal cells and disrupt the blood-brain barrier by directly damaging brain tissue (37). Leukocytes can penetrate a disrupted blood-brain barrier, which is linked to various stroke complications, including pathological cerebral edema, HT, and a decline in neurological function (38). Consequently, a rise in neutrophils is a crucial mediator of ischemic brain damage. More experimental data suggests that some specific lymphocyte subtypes, particularly CD4+ and CD8+ T cells, can release some cytotoxic chemicals and pro-inflammatory cytokines, even if the involvement of lymphocytes in ischemic brain injury is debatable. However, according to other research, lymphocytes are the primary cerebral protective immunomodulators following AIS and play a critical role in inflammation-induced neuroprotection (39). Second, after an ischemic stroke, platelets encourage brain damage (40–42). In mouse experiments, Ischemia-reperfusion in the brain causes platelet necrosis, which regulates injurious neutrophil recruitment and platelet-neutrophil aggregate formation and reduces cerebral blood flow (41). Platelets aggregate with circulating leukocytes when inflammation is activated *via* direct receptor-ligand interactions, activating platelet function and changing endothelial cell properties (43).

Up to now, some studies have reported the value of SII in cerebrovascular diseases and the association between high SII levels and clinical outcomes. Chu et al. (14) showed that SII increases dynamically from the onset of symptoms in AIS patients eligible for thrombolytic therapy. Higher levels of SII indicate more in-hospital complications and worse short-term prognoses. Another study from America demonstrated that in patients with supratentorial spontaneous ICH, the early SII was an independent predictor of poor outcomes at discharge (15). Hou et al. (9) investigated SII’s effect on stroke severity and found that SII was independently associated with stroke severity. Li et al. (16) demonstrated that SII, particularly in the acute phase (day 1), is highly correlated with 90-day functional outcomes in ICH patients. This index can be used to predict the prognosis. Weng et al. (17) found similar results to Li et al. and Hou et al. in AIS patients treated with IVT. In 2021, Yang et al. (18) conducted a more in-depth and detailed study, and the results showed that in patients with acute ischemia of the anterior circulation due to significant artery atherosclerosis, higher SII was associated with a greater risk of HT, particularly in artery-to-artery embolism and *in situ* thrombosis. This is the first study on HT. Then Yang et al. (27) and Yi et al. (19) continued to study this. Their findings demonstrated that admission SII is positively associated with HT in AIS due to large vessel occlusion patients treated with EVT, and higher SII meant more risk of HT. Besides, a study by Yi et al. (19) showed that a reduction in SII after mechanical thrombectomy (MT) was associated with favorable clinical outcomes. SII represents potential prognostic factors in patients undergoing MT for large artery occlusion. Acar et al. (20) considered SII a potential indicator to predict unsuccessful cerebral reperfusion and unfavorable clinical outcomes for patients with AIS undergoing EVT. Nevertheless, our analysis showed that no significant influence of SII for recanalization was observed. Further investigations were required. Adiguzel et al. (21) showed that significant variations of SII were observed during the first two weeks following the stroke. However, due to age and post-treatment clinical stroke severity, the discriminative capacity of these changes was limited. An investigation by Chen et al. (22) divided into two groups, one for in-hospital ischemic stroke (IHIS) and the other for out-of-hospital ischemic stroke (OHIS), and their study found that IHIS patients had more complicated clinical features, higher levels of SII and higher rates of mortality than OHIS patients. The underlying significance of the study was that IHIS patients should be paid more attention to in clinical practice. Hsu et al. (23) thought higher SII was associated with larger ICH volumes, poorer initial Glasgow Coma Scale, and unfavorable outcomes but was not an independent prognostic predictor. Furthermore, the association of SII and hematoma expansion in ICH patients requires further in-depth investigation. Huang et al. (10) further identified that higher SII meant a greater risk of stroke severity and adverse clinical outcomes after AIS. Ji et al. (24) identified elevated SII as related to malignant brain edema after EVT. Wang et al. (25) findings further affirmed that SII was closely associated with the short- and long-term prognosis of patients with AIS, and higher SII were more likely to have poor outcomes. Wu et al. (26) elevated SII increased the rate of 30-day all-cause mortality as an available index to elucidate the role of thrombocytosis, inflammation, and immunity interaction in developing AIS. Zhou et al. (28) obtained similar findings to previous studies, and a nomogram based on the SII could predict the risk of adverse outcomes in patients with AIS with an accuracy of 80.2%.

SII is not only associated with functional prognosis but also with psychic disorders. Hu et al. (44) conducted a study based on a prospective stroke cohort, and their findings showed that increased SII, especially SII at admission, is significantly correlated with post-stroke depression. The findings may provide some diagnostic clues to determine the early discovery of post-stroke depression. Nevertheless, whether SII is correlated with other psychic disorders secondary to stroke or not, comparative studies are urgent, and this may be the point of future research. Another study by Li et al. (45) demonstrated that the SII was not associated with neurological recovery at three months. In contrast, a low neutrophil-to-lymphocyte ratio was an independent factor for predicting neurological recovery three months after stroke. Topcuoglu et al. (46) considered that SII only had moderate utility and was far from perfection. In patients with HT, SII increased after bleeding occurred, and the admission values are not very valuable in this regard. Additionally, according to Li et al. (47) findings, the SII at admission is an independent predictor for the requirement of decompressive craniectomy (DC) in patients with large artery occlusion-AIS after MT. Based on the findings, the authors created a unique SII-based nomogram that assisted neurosurgeons in making prompt and sensible judgments about whether DC was required, potentially improving the prognosis of these patients. In elderly patients who underwent non-cardiac surgery, preoperative SII was a potential prognostic biomarker for predicting perioperative IS. SII > 583 was an independent risk factor for perioperative IS (48). In like manner, this finding may assist surgeons in avoiding severe complications and improving clinical outcomes.

By comprehensively and systematically reviewing the currently available literature, this may be the first meta-analysis assessing the relationship between SII and clinical outcomes of stroke patients. We obtained three significant findings by analysis: firstly, high SII is significantly associated with poor outcome, high mortality, and the incidence of HT; secondly, the SII of poor outcome, death, and moderate-to-severe stroke group was much higher than that of the excellent outcome, survival, and minor stroke group, respectively; thirdly, no significant influence was observed of SII for recanalization in patients with stroke who was undergoing operation procedure. Nevertheless, limited studies on some endpoints restricted the prevalence of our findings. Consequently, more studies on mortality, HT, recanalization, the difference of SII in the death/survival group, and stroke severity are urgent.

## Limitations

There are some limitations: first, other than randomized controlled trials, the majority of available studies are retrospective, and the study design may limit the evidence level of our findings; second, the majority of scholars are from China, and most of the participants are Chinese, too. Consequently, studies from other countries are required, as well as the other races of participants; third, the high heterogeneity of some endpoints influenced the robustness of our results. Despite these limitations, the preliminary findings of our meta-analysis may be useful to clinicians in making treatment decisions for stroke patients.

## Conclusion

To the best of our knowledge, this may be the first meta-analysis to look at the link between SII and clinical outcomes in stroke patients. The inflammatory response after a stroke is useful for immunoregulatory treatment. Stroke patients with high SII should be closely monitored, since this might be a viable treatment strategy for limiting brain damage after a stroke. As a result, research into SII and the clinical outcomes of stroke patients is crucial. Our preliminary findings may represent the clinical condition and aid clinical decision-makers. Nonetheless, further research is needed to better understand the utility of SII through dynamic monitoring. To generate more robust results, large-sample and multi-center research are required.

## Data availability statement

The original contributions presented in the study are included in the article/**Supplementary Material**. Further inquiries can be directed to the corresponding author.

## Author contributions

HY-W and YX-S developed the initial idea for this study. LZ-P developed and revised the search strategy. HY-W and YX-S formulated the study design. HY-W and YX-S contributed to the original draft. LZ-P was responsible for the revision of the draft. HY-W and YX-S contributed equally and are co-first authors. All authors contributed to the article and approved the submitted version.
