# Supplementary material for: Association of the systemic immune-inflammation index (SII) and clinical outcomes in patients with stroke: A systematic review and meta-analysis
Source: Front Immunol. 2022 Dec 15;13:1090305. doi: 10.3389/fimmu.2022.1090305 (PMC9797819; doi:10.3389/fimmu.2022.1090305)
Supplement: Supplementary file 1 [file DataSheet_1.pdf]

Supplementary Materials

Supplement to: Yong-Wei Huang, Xiao-Shuang Yin, Zong-Ping Li. Association of the systemic immune-inflammation index (SII) and clinical outcomes in patients with stroke: a systematic review and meta-analysis

Contents

Supplementary Table 1. PRISMA 2020 checklist.

Supplementary Table 2. Search strategy.

Supplementary Figure 1. Funnel plot of associated endpoints.

Supplementary Table 3. ROB assessment for the quality of studies in meta-analysis.

Supplementary Table 4. Confounding factors of adjusted ORs in each study.

Supplementary Table I. PRISMA 2020 checklist.

Section and Topic	Item #	Checklist item	Location where item is reported
**TITLE**			
Title	1	Identify the report as a systematic review.	page 1
**ABSTRACT**			
Abstract	2	See the PRISMA 2020 for Abstracts checklist.	page 2
**INTRODUCTION**			
Rationale	3	Describe the rationale for the review in the context of existing knowledge.	page 3
Objectives	4	provide an explicit statement of the objective(s) or question(s) the review addresses.	page 3
**METHODS**			
Eligibility criteria	5	Specify the inclusion and exclusion criteria for the review and how studies were grouped for the syntheses.	page 4
Information sources	6	Specify all databases, registers, websites, organisations, reference lists and other sources searched or consulted to identify studies. Specify the date when each source was last searched or consulted.	page 4
Search strategy	7	present the full search strategies for all databases, registers, and websites, including any filters and limits used.	page 4
Selection process	8	Specify the methods used to decide whether a study met the inclusion criteria of the review, including how many reviewers screened each record and each report retrieved, whether they worked independently, and if applicable, details of automation tools used in the process.	page 4
Data collection process	9	Specify the methods used to collect data from reports, including how many reviewers collected data from each report, whether they worked independently, any processes for obtaining or confirming data from study investigators, and if applicable, details of automation tools used in the process.	page 4
Data items	10a	List and define all outcomes for which data were sought. Specify whether all results that were compatible with each outcome domain in each study were sought (e.g. for all measures, time points, analyses), and if not, the methods used to decide which results to collect.	page 4
	10b	List and define all other variables for which data were sought (e.g. participant and intervention characteristics, funding sources). Describe any assumptions made about any missing or unclear information.	page 4
Study risk of bias assessment	11	Specify the methods used to assess risk of bias in the included studies, including details of the tool(s) used, how many reviewers assessed each study and whether they worked independently, and if applicable, details of automation tools used in the process.	page 4
Effect measures	12	Specify for each outcome the effect measure(s) (e.g., risk ratio, mean difference) used in the synthesis or presentation of results.	page 4-5
Synthesis methods	13a	Describe the processes used to decide which studies were eligible for each synthesis (e.g., tabulating the study intervention characteristics and comparing against the planned groups for each synthesis (item #5)).	page 4-5
	13b	Describe any methods required to prepare the data for presentation or synthesis, such as handling of missing summary statistics, or data conversions.	page 4-5
	13c	Describe any methods used to tabulate or visually display the results of individual studies and syntheses.	page 4-5
	13d	Describe any methods used to synthesize results and provide a rationale for the choice(s). If meta-analysis was performed, describe the model(s), method(s) to identify the presence and extent of statistical heterogeneity, and software package(s) used.	page 4-5
	13e	Describe any methods used to explore possible causes of heterogeneity among study results (e.g., subgroup analysis, meta-regression).	page 4-5
	13f	Describe any sensitivity analyses conducted to assess the robustness of the synthesized results.	page 4-5
Reporting bias assessment	14	Describe any methods used to assess the risk of bias due to missing results in a synthesis (arising from reporting biases).	page 4-5

Certainty assessment	15	Describe any methods used to assess certainty (or confidence) in the body of evidence for an outcome.	-
**RESULTS**			
Study selection	16a	Describe the results of the search and selection process, from the number of records identified in the search to the number of studies included in the review, ideally using a flow diagram.	page 5
	16b	Cite studies that might appear to meet the inclusion criteria, but which were excluded, and explain why they were excluded.	page 5
Study characteristics	17	Cite each included study and present its characteristics.	page 5
Risk of bias in studies	18	present assessments of risk of bias for each included study.	page 12
Results of individual studies	19	For all outcomes, present, for each study: (a) summary statistics for each group (where appropriate) and (b) an effect estimate and its precision (e.g. confidence/credible interval), ideally using structured tables or plots.	page 5-7
Results of syntheses	20a	For each synthesis, briefly summarise the characteristics and risk of bias among contributing studies.	page 8-12
	20b	present results of all statistical syntheses conducted. If meta-analysis was done, present for each the summary estimate and its precision (e.g. confidence/credible interval) and measures of statistical heterogeneity. If comparing groups, describe the direction of the effect.	page 8-12
	20c	present results of all investigations of possible causes of heterogeneity among study results.	page 9
	20d	present results of all sensitivity analyses conducted to assess the robustness of the synthesized results.	-
Reporting biases	21	present assessments of risk of bias due to missing results (arising from reporting biases) for each synthesis assessed.	page 11
Certainty of evidence	22	present assessments of certainty (or confidence) in the body of evidence for each outcome assessed.	None
**DISCUSSION**			
Discussion	23a	provide a general interpretation of the results in the context of other evidence.	page 12-14
	23b	Discuss any limitations of the evidence included in the review.	page 14
	23c	Discuss any limitations of the review processes used.	None
	23d	Discuss implications of the results for practice, policy, and future research.	page 14
**OTHER INFORMATION**			
Registration and protocol	24a	provide registration information for the review, including register name and registration number, or state that the review was not registered.	page 3
	24b	Indicate where the review protocol can be accessed, or state that a protocol was not prepared.	page 16
	24c	Describe and explain any amendments to information provided at registration or in the protocol.	page 3
Support	25	Describe sources of financial or non-financial support for the review, and the role of the funders or sponsors in the review.	page 15
Competing interests	26	Declare any competing interests of review authors.	page 15
Availability of data, code and other materials	27	Report which of the following are publicly available and where they can be found: template data collection forms; data extracted from included studies; data used for all analyses; analytic code; any other materials used in the review.	page 15

Supplemental Table 2. English databases (PubMed, Embase, Cochrane Library, Web of Science, and Scopus) and Chinese databases (CNKI, VIP, WanFang, and CBM) Search Strategy

Database	Search Strategy	Records
PubMed	(“systemic immune-inflammation index” OR “SII”) AND (“stroke”) **[All Fields]**	85
Embase	('systemic immune-inflammation index':ab,ti OR 'sii':ab,ti) AND 'stroke':ab,ti	108
Web of Science	(“systemic immune-inflammation index” OR “SII”) AND (“stroke”) **[All Fields]**	194
Scopus	('systemic immune-inflammation index':ab,ti OR 'sii':ab,ti) AND 'stroke':ab,ti	79
Cochrane Library	(“systemic immune-inflammation index”:ab,ti,kw OR “SII”:ab,ti,kw) AND (“stroke”:ab,ti,kw)	3
CNKI*	(主题=全身炎症免疫指数) AND (主题=卒中)	0
VIP*	(主题=全身炎症免疫指数) AND (主题=卒中)	1
WanFang*	(主题=全身炎症免疫指数) AND (主题=卒中)	1
CBM*	(主题=全身炎症免疫指数) AND (主题=卒中)	0
**Total**		469 + 2

* These databases were searched manually, and two articles met the included criteria.

Supplementary Figure 1. Funnel plot of associated endpoints.

Figure 1. The poor outcome between high SII and low SII group.

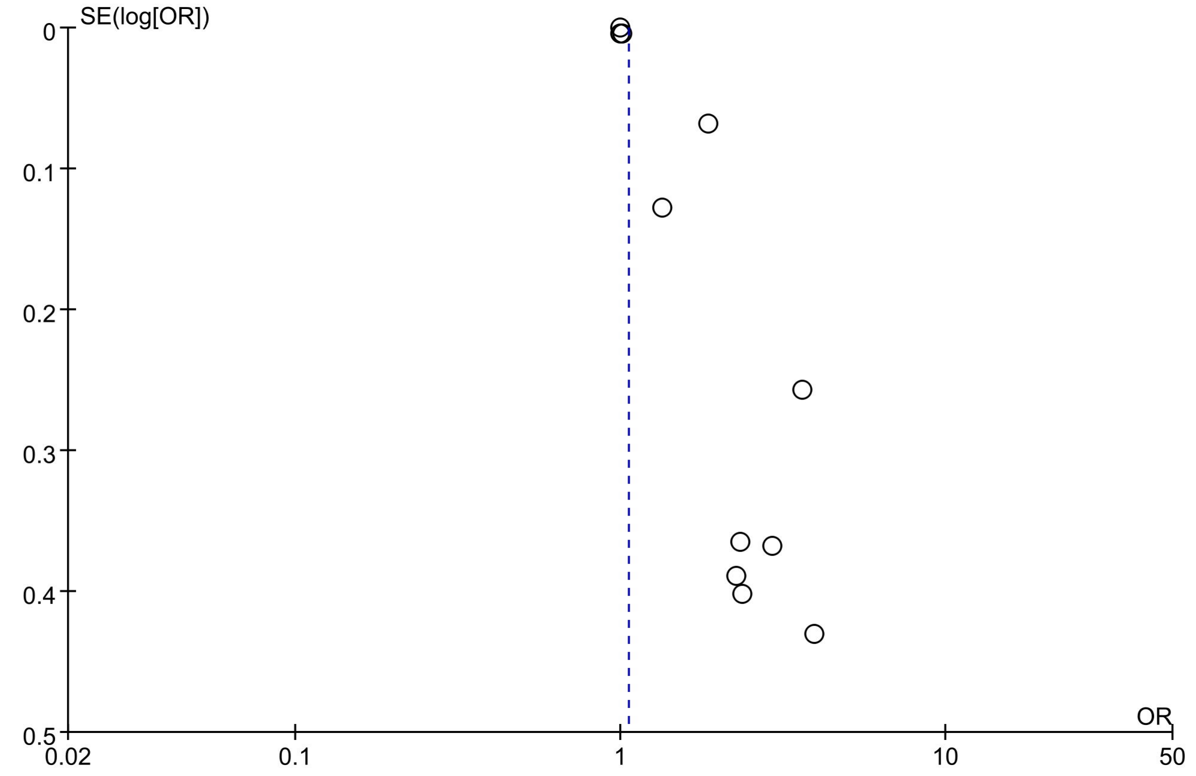

Figure 2. The mortality between high SII and low SII group.

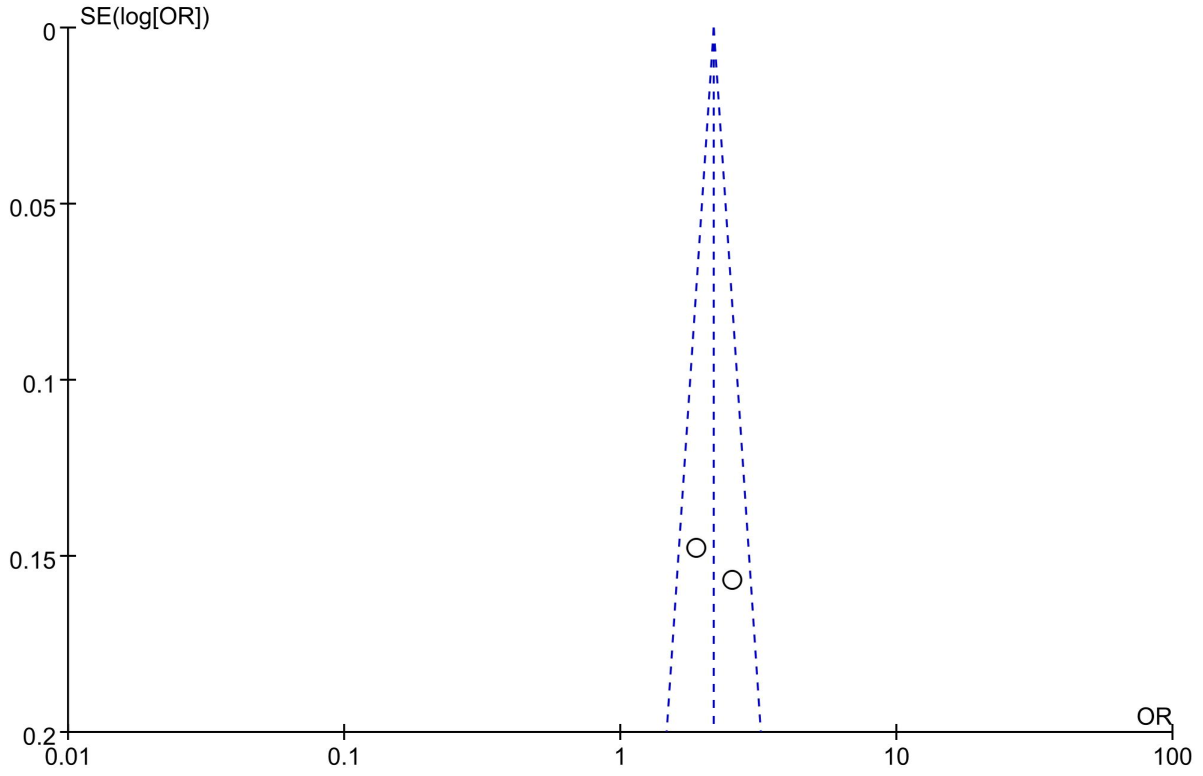

Figure 3. The HT between high SII and low SII group.

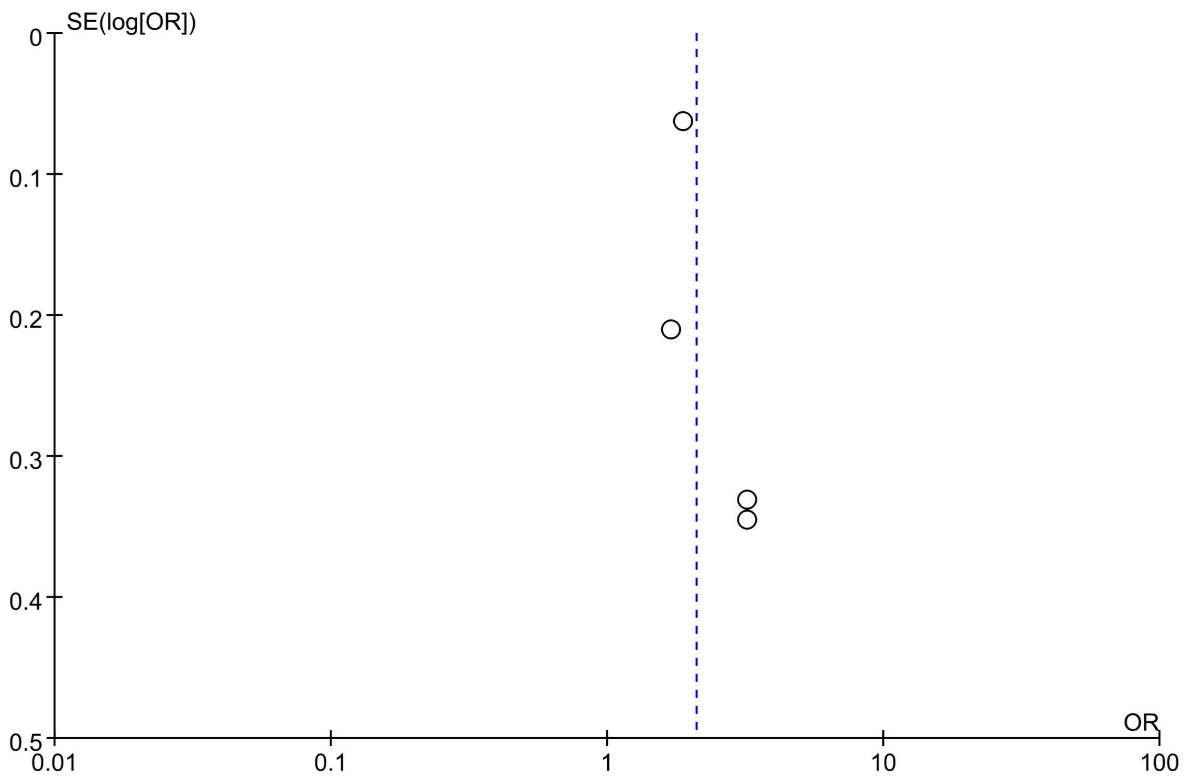

Figure 4. The recanalization between high SII and low SII group.

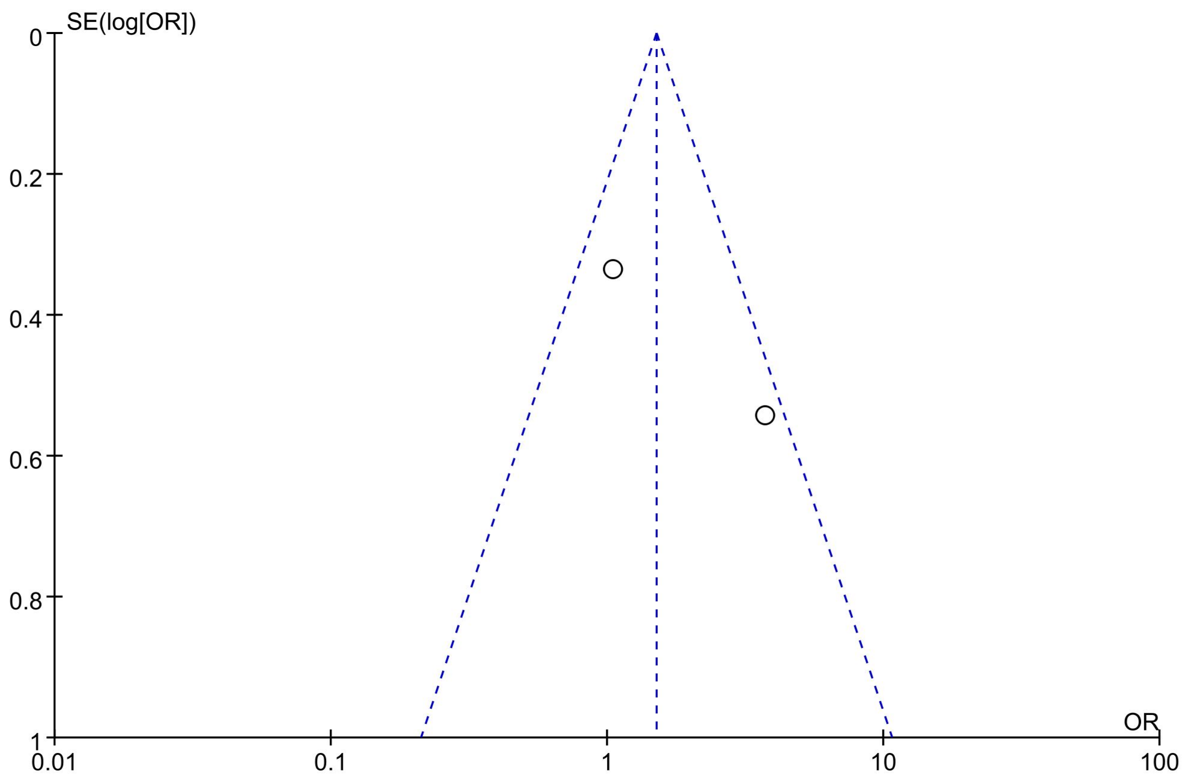

Figure 5. The subgroup analysis of poor outcome based on the **(A)** different countries, **(B)** different types of stroke, and **(C)** surgery intervention.

(A)

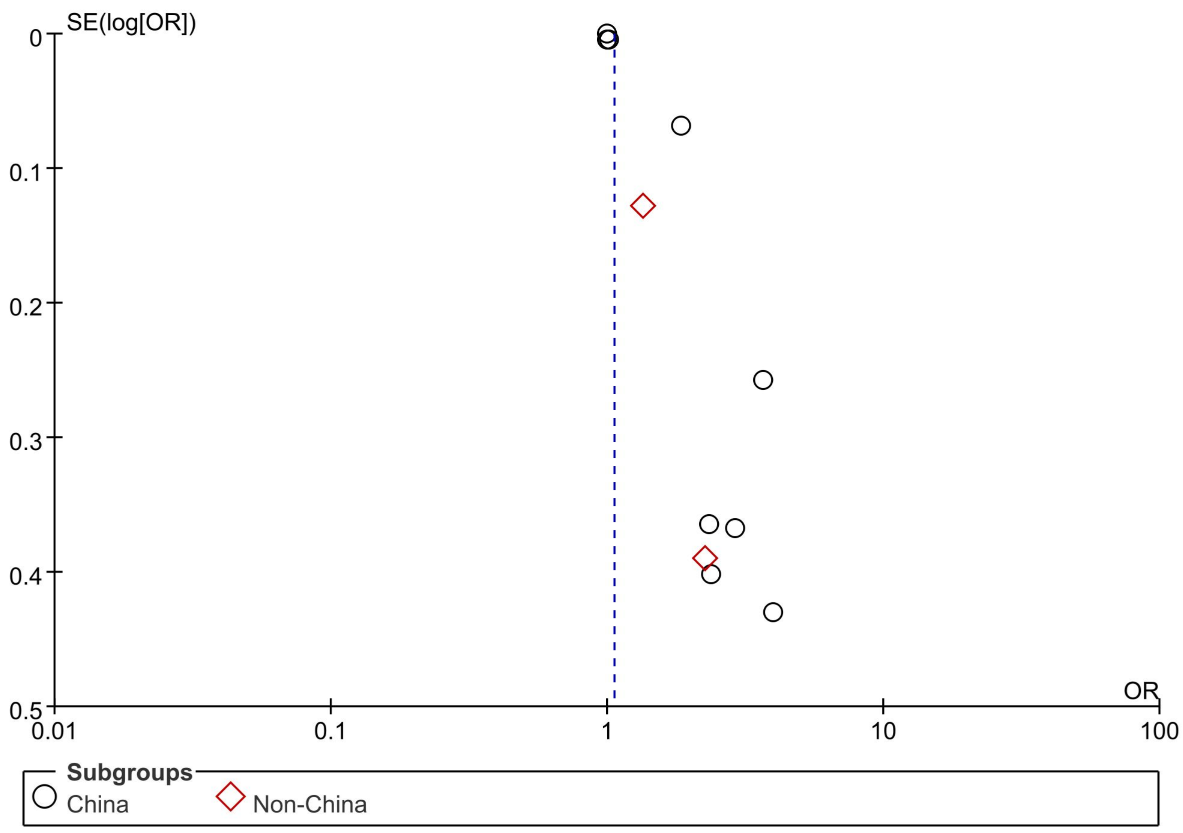

(B)

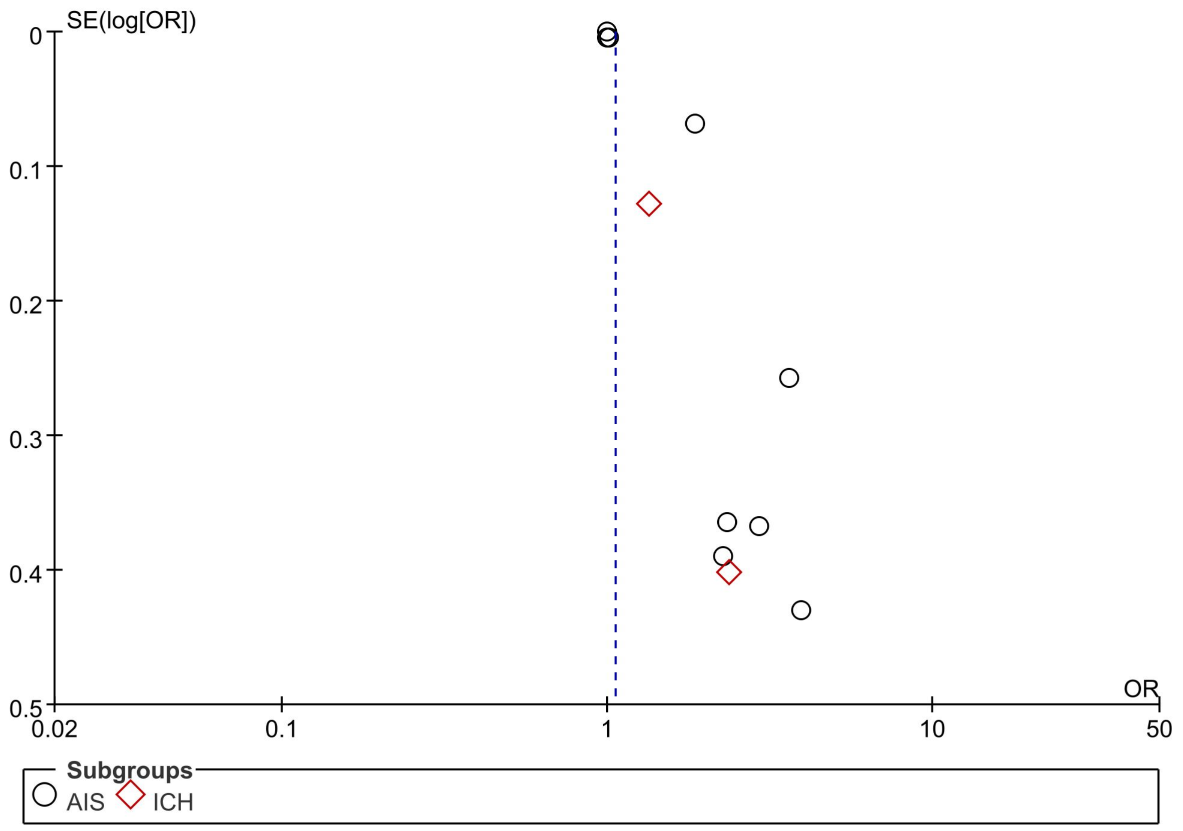

(C)

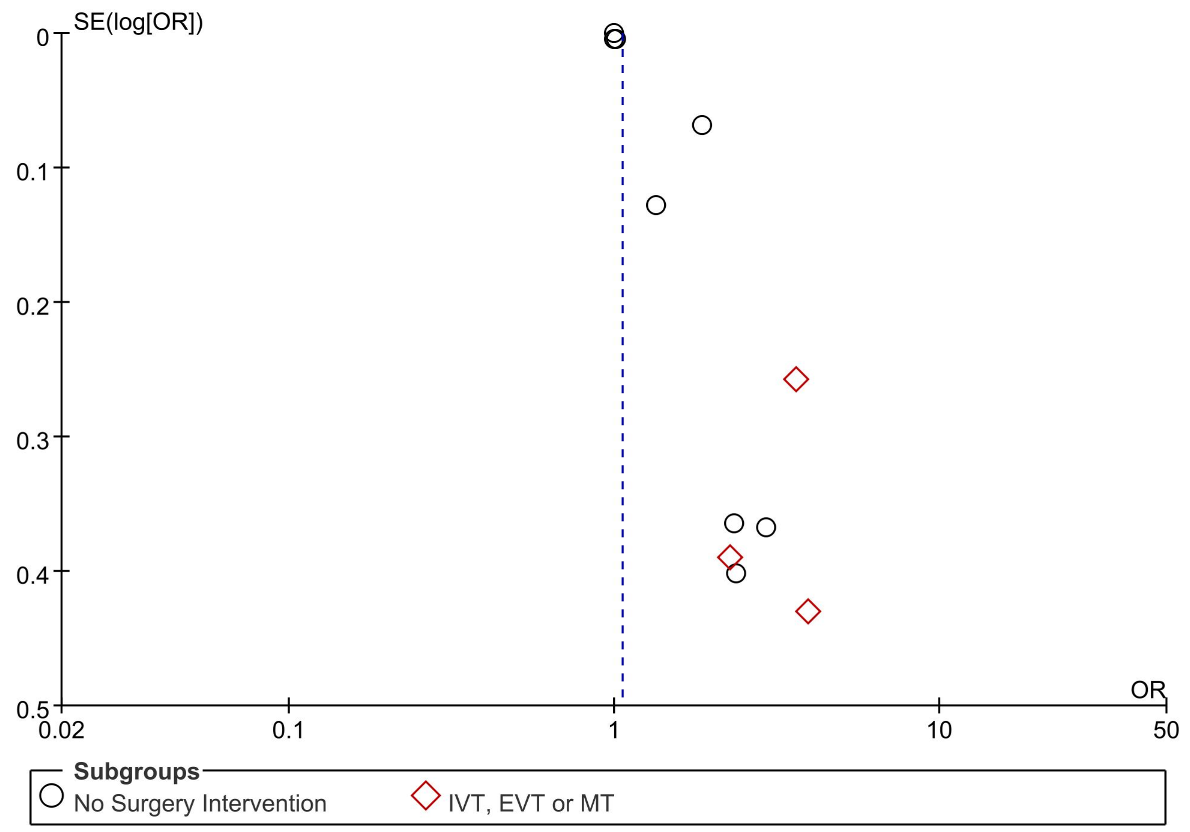

Figure 6. the SII level between the **(A)** poor outcome and good outcome group, **(B)** death and survival group, and **(C)** moderate-to-severe and minor stroke group.

(A)

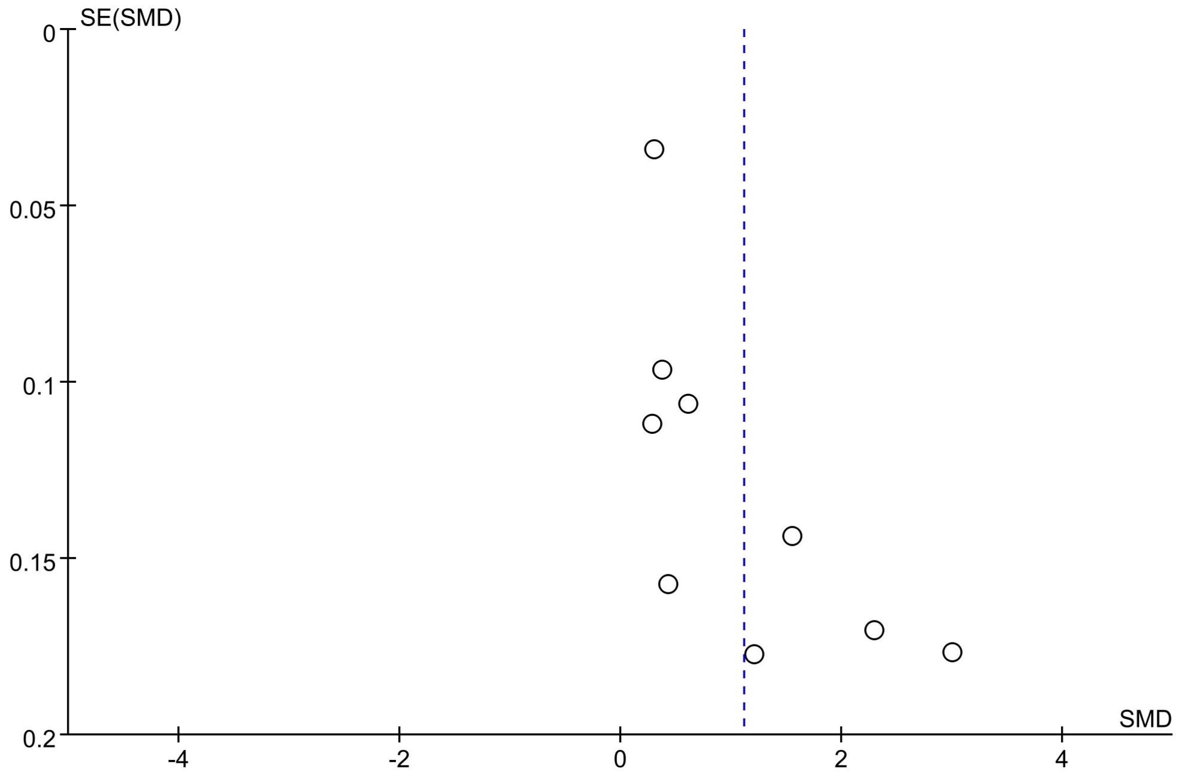

(B)

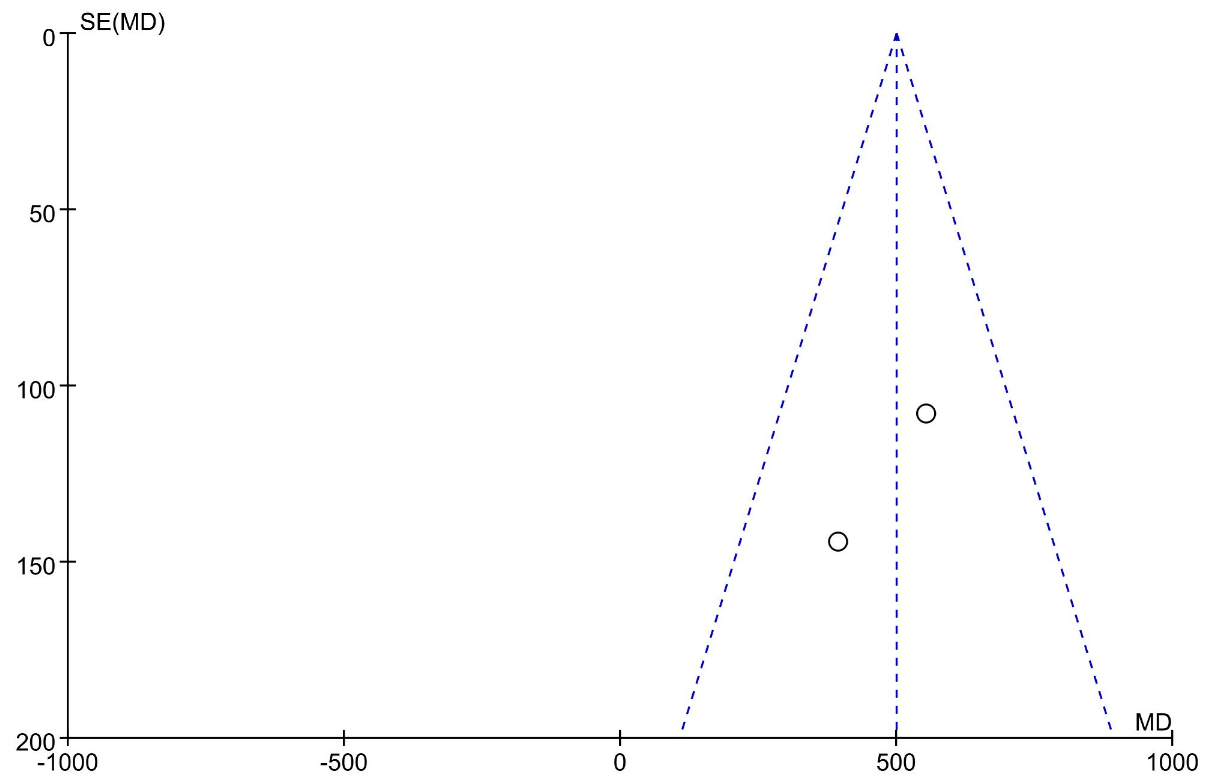
SE(MD)
0
50
100
150
200
-1000
-500
0
500
1000
MD

(C)

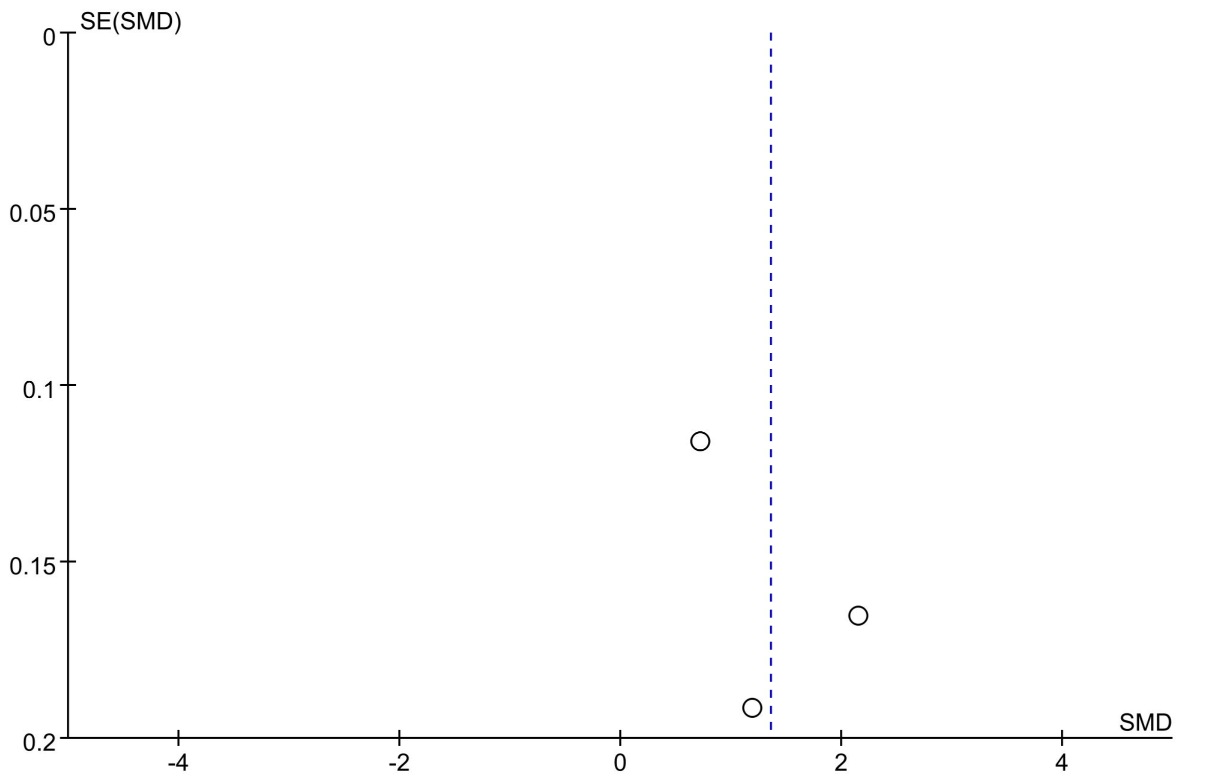
SE(SMD)
0
0.05
0.1
0.15
0.2
-4
-2
0
2
4
SMD

Supplemental Table 3. ROB assessment for the quality of studies in meta-analysis via NOS Scale.

Study	Year	Selection				Comparability		Exposure			Stars
		Q1	Q2	Q3	Q4	Q5	Q6	Q7	Q8	Q9	
Chu et al	2020	*	*	*	*	*	*	*	*	*	9
Trifan et al	2020	*	*	*	*	*	*	*	*	-	8
Hou et al	2021	*	*	*	*	*	*	*	*	*	9
Li et al	2021	*	*	*	*	-	*	*	*	-	7
Weng et al	2021	*	*	*	*	-	*	*	*	*	8
Yang et al	2021	*	*	*	*	*	*	*	*	*	9
Yi et al	2021	*	*	*	*	-	*	*	*	*	8
Acar et al	2022	*	*	*	*	*	*	*	*	-	8
Adiguzel et al	2022	*	*	*	*	-	*	*	*	-	7
Chen et al	2022	*	*	*	*	-	*	*	*	-	7
Hsu	2022	*	*	*	*	-	*	*	*	*	8
Huang et al	2022	*	*	*	*	*	*	*	*	*	9
Ji et al	2022	*	*	*	*	*	*	*	*	*	9
Wang et al	2022	*	*	*	*	-	*	*	*	-	7
Wu et al	2022	-	*	*	*	-	*	*	*	*	7
Yang et al	2022	*	*	*	*	-	*	*	*	*	8
Zhou et al	2022	*	*	*	*	*	*	*	*	*	9
Liu et al	2022	*	*	*	*	*	*	*	*	*	9
Zhu et al	2022	*	*	*	*	*	*	*	*	*	9

Q1 Is the case definition adequate?

Q2 Representativeness of the cases.

Q3 Selection of Controls.

Q4 Definition of Controls.

Q5 study controls for the most important factor.

Q6 study controls for any additional factor.

Q7 Ascertainment of exposure.

Q8 Same method of ascertainment for cases and controls.

Q9 Non-Response rate.

Supplementary Table 4. Confounding factors of adjusted ORs in each study.

Items	Author	Year	Adjusted ORs (95%CI)	Confounding factors mentioned or not
**Poor outcome**	Acar	2022	2.280 (1.060-4.880)	Age, Sex, Diabetes mellitus, Smoking, Prior stroke, Dyslipidemia, Hypertension, Obesity, Atrial fibrillation, Systolic blood pressure, Initial NIHSS score <10, ICA occlusion, Middle cerebral artery occlusion, Good collateral status, Prior use of antiplatelets, Symptom to puncture time, IVT, First-pass reperfusion, Serum glucose, GFR, hs-CRP.
	Huang	2022	2.350 (1.149-4.803)	SIRI, Hs-CRP, NIHSS.
	Ji	2022	3.639 (2.197-6.027)	Age, sex, Hypertension, Diabetes mellitus AF, Admission SBP, Admission NIHSS, Admission ASPECT, Collateral Grade, Occlusion site, mTICI, FBG.
	Li	2021	2.360 (1.090-5.260)	sex, age, admission Glasgow Coma Scale, logarithm intracerebral hematoma volume, intraventricular hematoma occurrence, hematoma location and craniotomy.
	Trifan	2020	1.340 (1.040-1.720)	GCS, DM, CKD, ICH volume and IVH extension
	Wang	2022	1.86 (1.63-2.13)	sex, age, smoking status, alcohol consumption, history of cerebral infarction, hypertension, atrial fibrillation, coronary heart disease, diabetes mellitus, WBC, FPG, LDL-C, Hcy, hs-CRP, hours of event onset, NIHSS score at onset, and Mrs score before onset ≥3.
	Weng	2021	3.953 (1.702-9.179)	age, current smoking, AF, prior stroke and baseline NIHSS score, hypertension, and diabetes.
	Zhou	2022	2.915 (1.42-5.985)	age, grade, NIHSS score, diabetes, smoking, hypertension,
	Liu[a]	2022	1.009 (1.002-1.023)	Sex, age, hypertension, diabetes, AF, fasting blood glucose, glycerin trilaurate,
	Liu[b]	2022	1.001 (0.982-1.001)	
	Zhu	2022	1.001 (1.000-1.002)	NIHSS, hs-CRP, hypertension, D-D
**HT**	Yang	2021	3.214 (1.633-6.326)	age, sex, SBP, NIHSS, anticoagulation, thrombolysis, Artery-to-artery embolization, In-situ thrombosis, Hypoperfusion, Branch atheromatous disease
	Yang	2022	3.23 (1.69-6.19)	baseline ASPECTS, the hyper-dense sign of MCA, PTR time, number of passes > 3, TOAST classification, age, baseline NIHSS, IVT.
	Yi	2022	1.7 (1.12-2.56)	Age, ASPECTS (>8), Initial NIHSS score (<8), First-pass reperfusion, Successful recanalization

				NLR (<3.7), LMR (≥2.2), PLR (<125), SIRI (<2.9)
	Liu	2022	1.874 (1.126-1.438)	Sex, age, hypertension, diabetes, AF, fasting blood glucose, glycerin trilaurate
